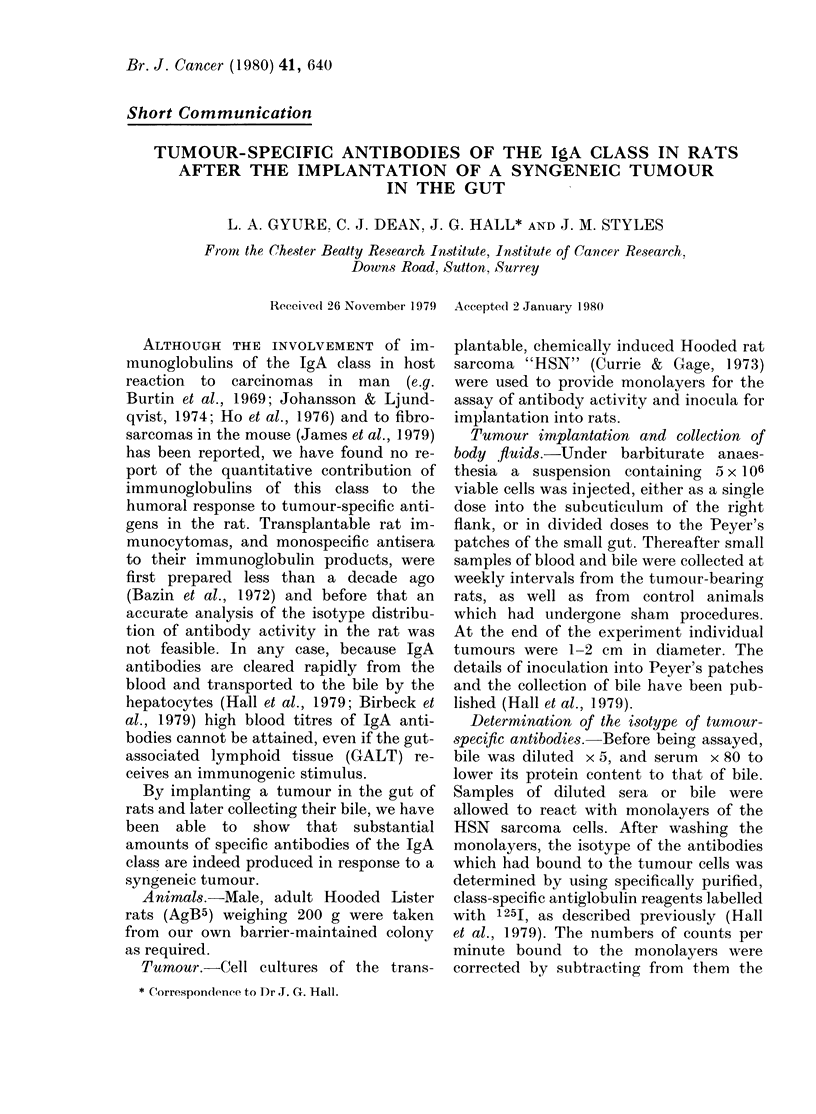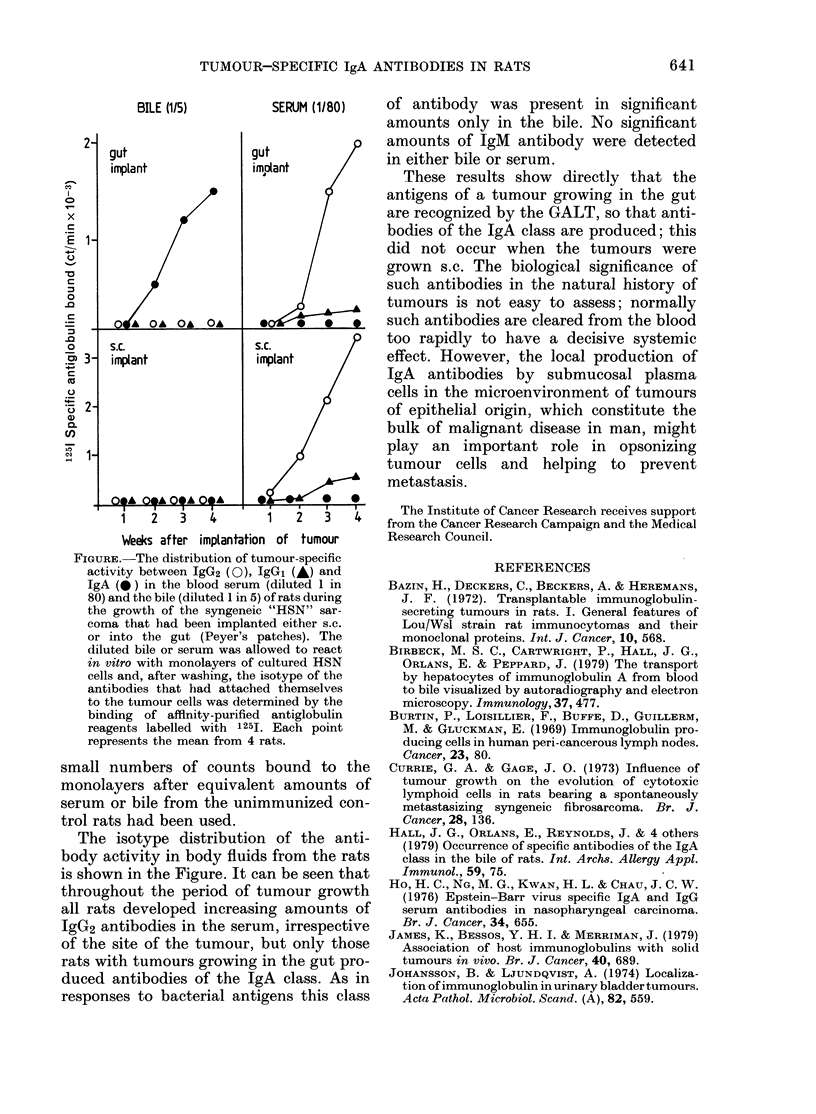# Tumour-specific antibodies of the IgA class in rats after the implantation of a syngeneic tumour in the gut.

**DOI:** 10.1038/bjc.1980.108

**Published:** 1980-04

**Authors:** L. A. Gyure, C. J. Dean, J. G. Hall, J. M. Styles


*Br. J. Cancer* (1980) **41**, 640

***Short Communication***

### TUMOUR-SPECIFIC ANTIBODIES OF THE IgA CLASS IN RATS AFTER THE IMPLANTATION OF A SYNGENEIC TUMOUR IN THE GUT

L. A. GYURE, C. J. DEAN, J. G. HALL* AND J. M. STYLES

*From the Chester Beatty Research Institute, Institute of Cancer Research, Downs Road, Sutton, Surrey*

Received 26 November 1979 Accepted 2 January 1980

ALTHOUGH THE INVOLVEMENT of immunoglobulins of the IgA class in host reaction to carcinomas in man (*e.g.* Burtin *et al.*, 1969; Johansson & Ljundqvist, 1974; Ho *et al.*, 1976) and to fibrosarcomas in the mouse (James *et al.*, 1979) has been reported, we have found no report of the quantitative contribution of immunoglobulins of this class to the humoral response to tumour-specific antigens in the rat. Transplantable rat immunocytomas, and monospecific antisera to their immunoglobulin products, were first prepared less than a decade ago (Bazin *et al.*, 1972) and before that an accurate analysis of the isotype distribution of antibody activity in the rat was not feasible. In any case, because IgA antibodies are cleared rapidly from the blood and transported to the bile by the hepatocytes (Hall *et al.*, 1979; Birbeck *et al.*, 1979) high blood titres of IgA antibodies cannot be attained, even if the gut-associated lymphoid tissue (GALT) receives an immunogenic stimulus.

By implanting a tumour in the gut of rats and later collecting their bile, we have been able to show that substantial amounts of specific antibodies of the IgA class are indeed produced in response to a syngeneic tumour.

*Animals*.—Male, adult Hooded Lister rats ($AgB^5$) weighing 200 g were taken from our own barrier-maintained colony as required.

*Tumour*.—Cell cultures of the transplantable, chemically induced Hooded rat sarcoma "HSN" (Currie & Gage, 1973) were used to provide monolayers for the assay of antibody activity and inocula for implantation into rats.

*Tumour implantation and collection of body fluids*.—Under barbiturate anaesthesia a suspension containing $5 \times 10^6$ viable cells was injected, either as a single dose into the subcuticulum of the right flank, or in divided doses to the Peyer's patches of the small gut. Thereafter small samples of blood and bile were collected at weekly intervals from the tumour-bearing rats, as well as from control animals which had undergone sham procedures. At the end of the experiment individual tumours were 1–2 cm in diameter. The details of inoculation into Peyer's patches and the collection of bile have been published (Hall *et al.*, 1979).

*Determination of the isotype of tumour-specific antibodies*.—Before being assayed, bile was diluted $\times 5$, and serum $\times 80$ to lower its protein content to that of bile. Samples of diluted sera or bile were allowed to react with monolayers of the HSN sarcoma cells. After washing the monolayers, the isotype of the antibodies which had bound to the tumour cells was determined by using specifically purified, class-specific antiglobulin reagents labelled with $^{125}I$, as described previously (Hall *et al.*, 1979). The numbers of counts per minute bound to the monolayers were corrected by subtracting from them the

* Correspondence to Dr J. G. Hall.

FIGURE.—The distribution of tumour-specific activity between $IgG_2$ (○), $IgG_1$ (▲) and IgA (●) in the blood serum (diluted 1 in 80) and the bile (diluted 1 in 5) of rats during the growth of the syngeneic "HSN" sarcoma that had been implanted either s.c. or into the gut (Peyer's patches). The diluted bile or serum was allowed to react *in vitro* with monolayers of cultured HSN cells and, after washing, the isotype of the antibodies that had attached themselves to the tumour cells was determined by the binding of affinity-purified antiglobulin reagents labelled with $^{125}I$. Each point represents the mean from 4 rats.

small numbers of counts bound to the monolayers after equivalent amounts of serum or bile from the unimmunized control rats had been used.

The isotype distribution of the antibody activity in body fluids from the rats is shown in the Figure. It can be seen that throughout the period of tumour growth all rats developed increasing amounts of $IgG_2$ antibodies in the serum, irrespective of the site of the tumour, but only those rats with tumours growing in the gut produced antibodies of the IgA class. As in responses to bacterial antigens this class of antibody was present in significant amounts only in the bile. No significant amounts of IgM antibody were detected in either bile or serum.

These results show directly that the antigens of a tumour growing in the gut are recognized by the GALT, so that antibodies of the IgA class are produced; this did not occur when the tumours were grown s.c. The biological significance of such antibodies in the natural history of tumours is not easy to assess; normally such antibodies are cleared from the blood too rapidly to have a decisive systemic effect. However, the local production of IgA antibodies by submucosal plasma cells in the microenvironment of tumours of epithelial origin, which constitute the bulk of malignant disease in man, might play an important role in opsonizing tumour cells and helping to prevent metastasis.

The Institute of Cancer Research receives support from the Cancer Research Campaign and the Medical Research Council.

REFERENCES

BAZIN, H., DECKERS, C., BECKERS, A. & HEREMANS, J. F. (1972). Transplantable immunoglobulin-secreting tumours in rats. I. General features of Lou/Wsl strain rat immunocytomas and their monoclonal proteins. *Int. J. Cancer*, **10**, 568.

BIRBECK, M. S. C., CARTWRIGHT, P., HALL, J. G., ORLANS, E. & PEPPARD, J. (1979) The transport by hepatocytes of immunoglobulin A from blood to bile visualized by autoradiography and electron microscopy. *Immunology*, **37**, 477.

BURTIN, P., LOISILLIER, F., BUFFE, D., GUILLERM, M. & GLUCKMAN, E. (1969) Immunoglobulin producing cells in human peri-cancerous lymph nodes. *Cancer*, **23**, 80.

CURRIE, G. A. & GAGE, J. O. (1973) Influence of tumour growth on the evolution of cytotoxic lymphoid cells in rats bearing a spontaneously metastasizing syngeneic fibrosarcoma. *Br. J. Cancer*, **28**, 136.

HALL, J. G., ORLANS, E., REYNOLDS, J. & 4 others (1979) Occurrence of specific antibodies of the IgA class in the bile of rats. *Int. Archs. Allergy Appl. Immunol.*, **59**, 75.

HO, H. C., NG, M. G., KWAN, H. L. & CHAU, J. C. W. (1976) Epstein–Barr virus specific IgA and IgG serum antibodies in nasopharyngeal carcinoma. *Br. J. Cancer*, **34**, 655.

JAMES, K., BESSOS, Y. H. I. & MERRIMAN, J. (1979) Association of host immunoglobulins with solid tumours *in vivo*. *Br. J. Cancer*, **40**, 689.

JOHANSSON, B. & LJUNDQVIST, A. (1974) Localization of immunoglobulin in urinary bladder tumours. *Acta Pathol. Microbiol. Scand.* (A), **82**, 559.